# Supplementary material for: The causal relationship between the human gut microbiota and pyogenic arthritis: a Mendelian randomization study
Source: Front Cell Infect Microbiol. 2024 Nov 26;14:1452480. doi: 10.3389/fcimb.2024.1452480 (PMC11629706; doi:10.3389/fcimb.2024.1452480)
Supplement: Supplementary file 1 [file DataSheet1.zip › mendelian test/ebi-a-GCST90027837.csv_leave_one_out.pdf]

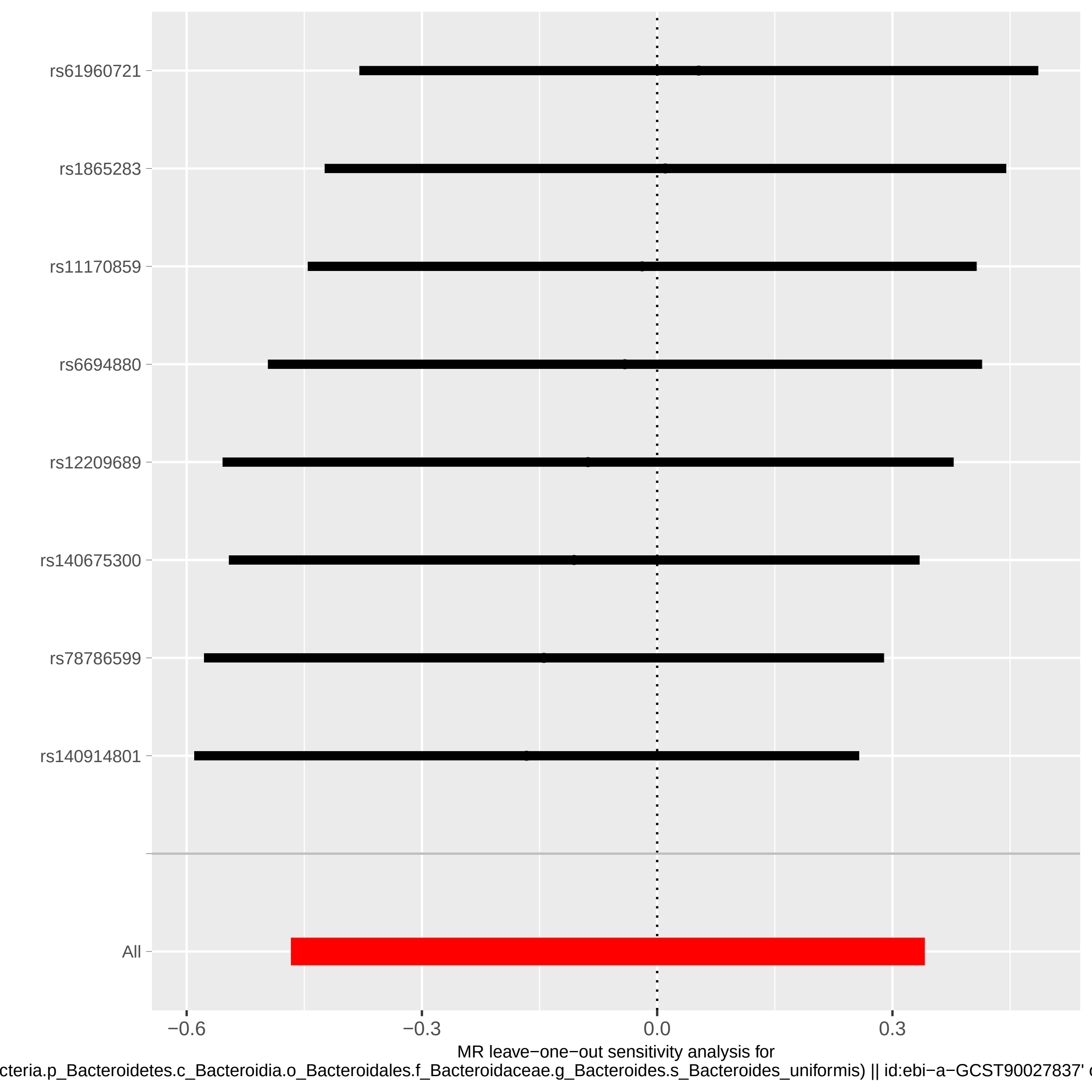

rs61960721
rs1865283
rs11170859
rs6694880
rs12209689
rs140675300
rs78786599
rs140914801
All
−0.6
−0.3
0.0
0.3
MR leave−one−out sensitivity analysis for
cteria.p_Bacteroidetes.c_Bacteroidia.o_Bacteroidales.f_Bacteroidaceae.g_Bacteroides.s_Bacteroides_uniformis) || id:ebi−a−GCST90027837' o
